# Study on the Hierarchical Predictive Control of Semiconductor Silicon Single Crystal Quality Based on the Soft Sensor Model

**DOI:** 10.3390/s23052830

**Published:** 2023-03-05

**Authors:** Yin Wan, Ding Liu, Jun-Chao Ren, Shi-Hai Wu

**Affiliations:** 1 School of Automation and Information Engineering, Xi’an University of Technology, Xi’an 710048, China; yinwan690212@163.com (Y.W.); renjc425x@163.com (J.-C.R.); wushihai0206@163.com (S.-H.W.); 2 Shaanxi Key Laboratory of Complex System Control and Intelligent Information Processing, Xi’an University of Technology, Xi’an 710048, China

**Keywords:** SSC quality monitoring and control, hierarchical predictive control, MPC, PID, soft sensor model

## Abstract

Silicon single crystal (SSC) quality monitoring and control has been a hot research topic in the field of the Czochralski crystal growth process. Considering that the traditional SSC control method ignores the crystal quality factor, this paper proposes a hierarchical predictive control strategy based on a soft sensor model for online control of SSC diameter and crystal quality. First, the proposed control strategy considers the V/G variable (V is the crystal pulling rate, and G is the axial temperature gradient at the solid–liquid interface), a factor related to crystal quality. Aiming at the problem that the V/G variable is difficult to measure directly, a soft sensor model based on SAE-RF is established to realize the online monitoring of the V/G variable and then complete hierarchical prediction control of SSC quality. Second, in the hierarchical control process, PID control of the inner layer is used to quickly stabilize the system. Model predictive control (MPC) of the outer layer is used to handle system constraints and enhance the control performance of the inner layer. In addition, the SAE-RF-based soft sensor model is used to monitor the crystal quality V/G variable online, thereby ensuring that the output of the controlled system meets the desired crystal diameter and V/G requirements. Finally, based on the industrial data of the actual Czochralski SSC growth process, the effectiveness of the proposed crystal quality hierarchical predictive control method is verified.

## 1. Introduction

**Figure 1:**
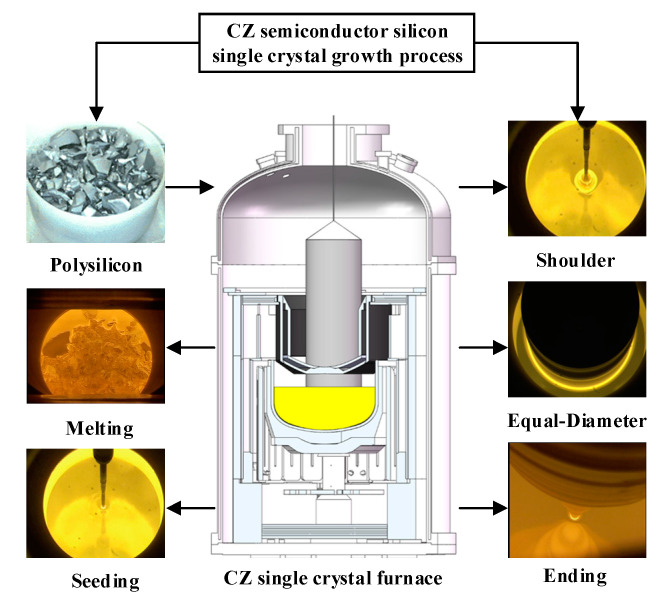
Schematic diagram of the CZ silicon single crystal growth process.

The information technology industry and the green new energy industry are the two pillar industries for the development of human society. Semiconductor silicon single crystal (SSCs) are the mainstream basic material for the two pillar industries. Therefore, the quality of SSC is crucial to the rapid development of the integrated circuit (IC) industry. In the early 1970s, researchers proposed the direction of “defect elimination” to obtain better quality crystals [1]. Currently, the Czochralski (CZ) crystal growth process is the main method for producing SSC, as shown in Figure 1. The improvement of crystal quality focuses on monitoring and controlling the SSC growth process in the CZ single crystal furnace [2]. With the rapid development of IC technology, it puts forward higher requirements on the diameter size and crystal quality of semiconductor SSCs. Furthermore, the strong nonlinearity, strong coupling, and model uncertainty of the CZ SSC growth process (CZ-SSCGP) make it difficult for traditional model-based control methods to achieve satisfactory production targets [3]. Therefore, it is of great practical value and significance to study the quality control of semiconductor SSCs.

In recent decades, many scholars have devoted themselves to CZ-SSCGP control and achieved research results [4,5,6]. Irizarry et al. [7] designed a dual-model predictive controller, an MPC controller used to control pulling speed, and a predictive controller used to control the crystal diameter. Ren et al. [8] proposed a crystal diameter adaptive NMPC method based on a hybrid integrated prediction model for the crystal diameter control problem, which can not only achieve accurate control of the crystal diameter but also effectively suppress the effects of external disturbances and time lag variations, with good control performance, as well as engineering application prospects. Rahmanpour et al. [9] established a simplified kinetic model of the CZ process based on quasi-steady-state simulation results and designed two mutually collaborative nonlinear MPC controllers based on the model for the crystal diameter and melt temperature in the batch process to achieve robust and effective control of the CZ process. Liu et al. [10] realized nonlinear generalized predictive control of the crystal diameter during the growth of CZ-Si crystal based on the stacked sparse autoencoder network. Wan et al. [11] used the V/G value as the crystal quality measurement standard for the first time and introduced MPC control to realize the control of crystal size and micro-defects.

The goal of preparing SSC by the CZ process is to grow crystals with the advantages of equal diameter, fewer impurities and fewer defects [12,13]. However, due to industry or technical method limitations, people focus more on how to achieve accurate control of crystal shape size and thermal field temperature, and research on crystal quality is more in the stage of numerical simulation. Few people study how to implement quality monitoring. Therefore, how to control the original point defects in the crystal, increase the integrity, and ensure the quality of the crystal while controlling the crystal diameter has become a research difficulty in the control of the CZ-SSCGP.

The preparation and defect control of large-size semiconductor SSCs has been a hot research direction in the silicon material industry in recent years [14,15,16,17,18]. SSC preparation technology in some developed countries is aimed in two directions. For example, the “super silicon” project carried out by a Japanese research team with the support of the state mainly studied large-diameter SSCs [19]. Under normal circumstances, the concentration of point defects inside the crystal is not measurable. In 1982, Voronkov used a reasonable mathematical model to describe the behavior of point defects existing in the crystal. The V/G theory proposed by Voronkov assumes that the crystal contains only point defects at high temperatures—vacancies or self-interstitial [20]. According to the V/G theory proposed by Voronkov, when V/G lies between 1 and 2 (unit: cm^2^ K^−1^ min^−1^), the concentration of the two atoms in the crystal is close to each other. Then, during the post-heat treatment process, the two atoms are rapidly compounded, and there are almost no remaining atoms; thus, the low-defect, dislocation-free crystal with perfect compounding for IC manufacturing can be obtained [21]. When V/G exceeds this range, the concentration of vacancy atoms within the crystal in the initial stage is high. When the temperature decreases, the two atoms begin to compound, and the excess vacancy atoms aggregate into defects dominated by vacancy atoms during the cooling of SSCs. When V/G is lower than this range, the concentration of interstitial atoms within the crystal is higher in the initial stage. When the temperature decreases, the two atoms compound and the excess interstitial atoms aggregate into defects dominated by interstitial atoms during the cooling of the SSC. At present, the control of defects in semiconductor SSCs is generally achieved by setting appropriate process parameters or adding cold traps.

To address the above-mentioned problems in the CZ-SSCGP, this paper considers the establishment of a soft sensor model for the crystal quality-related variable V/G. Based on this, a hierarchical prediction control strategy for SSC quality in the CZ-SSCGP is proposed. This method aims to optimize the control output performance of the system and realize the online monitoring of crystal diameter and quality to meet the actual industrial production goal. The main contributions of this paper are as follows:

(1) For semiconductor SSC quality monitoring, we developed a soft sensor model based on SAE-RF to complete V/G prediction related to crystal quality in CZ-SSCGP. This method solves the difficulty that traditional crystal quality cannot be directly monitored online.
(2) In this paper, we propose a hierarchical predictive control method for SSC quality, including an external MPC control layer, an internal PID control layer, and a V/G soft sensor monitoring model, to realize online monitoring of crystal quality.
(3) Various simulation results show that the proposed SAE-RF-based V/G soft sensor model has an accurate crystal quality V/G prediction performance. In addition, the proposed hierarchical predictive control of SSC quality can achieve precise control of crystal diameter while also ensuring that crystal quality meets actual industrial requirements.

The rest of this paper is organized as follows. Section 2 briefly introduces the CZ-SSCGP and the hierarchical predictive control strategy for SSC quality. Section 3 details the hierarchical predictive control method for SSC quality based on the soft sensor model. Specifically, the outer layer MPC control algorithm and the inner layer PID control algorithm are given. Various experimental results and discussions are presented in Section 4. Finally, Section 5 presents the conclusions of our research work.

## 2. Cz-SSCGP and Quality Control Problem Description

### 2.1. A Brief Description of CZ-SSSCGP

**Figure 2:**
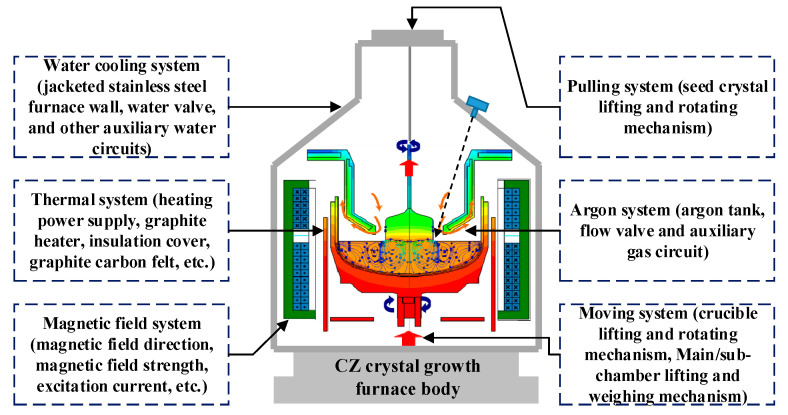
Block diagram of the CZ silicon single crystal growth environment.

The CZ-SSCGP is complex and involves several process variables. To ensure the stable operation of the SSC growth process and SSC quality and to meet the requirements of the IC industry, the primary task is to ensure that the crystal diameter variable remains fixed or does not fluctuate significantly during the equal-diameter growth process. Crystal diameter is a key controlled variable in the CZ-SSCGP, and its tuning process depends on manipulated variables, such as heater power and pulling rate [2]. Therefore, more attention has been paid to research on SSC growth control. Here, complex SSC growth characteristics such as nonlinearity and strong coupling inherent in the CZ process are shown in Figure 2.

Facing the complexity of the SSC growth environment, it is difficult for the previous control methods to ensure that the V/G related to crystal quality is within the desired control range. Therefore, to ensure that the large-size SSC produced in the industry meets the quality requirements of equal diameter, low defects, and no dislocations, it is necessary to develop a new control strategy to control the crystal diameter and quality. To this end, this paper introduces a soft sensor modeling method for crystal quality. Based on this, a hierarchical predictive control strategy based on the idea of Identification for Control (I4C) is proposed to realize online monitoring and control of crystal quality V/G and crystal diameter.

### 2.2. A Hierarchical Predictive Control Strategy for Semiconductor SSC

**Figure 3:**
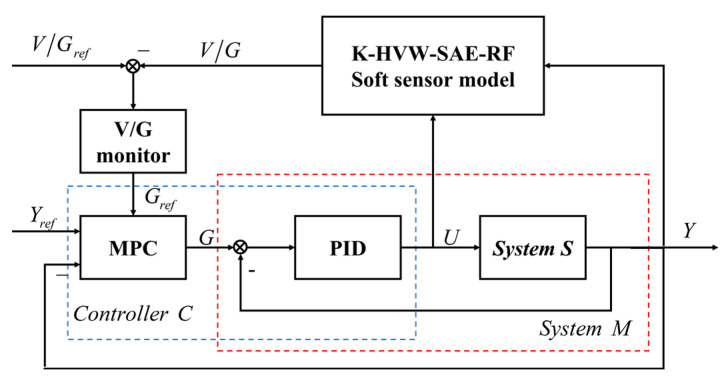
Hierarchical predictive control structure diagram.

The control of the crystal diameter has always been the core of the CZ-SSCGP. Precise diameter control can not only improve the utilization rate of SSC ingots and reduce costs, but the reduction of its fluctuation range is also an indispensable factor for the reduction of crystal defects. At present, PID control is commonly used in actual industrial production, but the CZ-SSCGP is a nonlinear and large delay process. Traditional PID control cannot achieve precise diameter tracking control nor can it handle constraints. As a nonlinear control method, MPC is widely used in SSC diameter control. However, in traditional MPC, the closed-loop control performance of the system can be optimal only when the plant model is very accurate. Otherwise, system robustness needs to be improved at the expense of control performance. In other words, the control performance of the MPC depends on the accuracy of the predictive model [22,23,24,25,26]. In this paper, by directly considering the control target in the model learning stage, according to the I4C idea, the predictive model is used as a design parameter to iteratively optimize the internal controller to find the best MPC predictive model; thus, a hierarchical predictive control structure with a soft sensor model, as shown in Figure 3, is proposed [27]. However, the MPC currently needs further exploration in coping with the uncertainty of the discriminative model. In this regard, a series of robust controllers based on sliding film control studied by Gohari et al. [22,23] performed well in coping with system uncertainty. However, these methods also have their own limitations, such as the improvement of controller robustness, which will sacrifice the system control performance to some extent. Therefore, to solve this problem and resolve the conflict between MPC controller robustness and system control performance, this paper finds the best MPC prediction model by directly considering the control objective in the model learning stage and iteratively optimizing the prediction model as design parameters with the internal controller according to the I4C idea [24]. Therefore, a hierarchical control structure considering soft measurement theory is proposed, as shown in Figure 3. The controller designed in this paper can adapt to the change in operating conditions and determine the system model adaptively, and it can suppress the system disturbance very quickly to ensure the smooth operation of the system. It is noteworthy that the proposed control method does not adjust the controller to handle uncertainties but rather adjusts the learning process to select a predictive model to provide the best crystal diameter and quality coordinated control performance.

## 3. A Hierarchical Control Method Based on the Soft Sensor Mode

### 3.1. Inner Controller Design

In hierarchical predictive control based on a soft sensor model, inner control is used to improve the system’s rapidity, and the control method chosen by the inner controller can guarantee the system’s rapidity. Here, we used the PID method to design the inner controller. It has been widely used because of its simple control rules, easy implementation and non-dependence on mathematical models. According to the principle of PID control, PID has three parameters $K_{p}$, $K_{i}$, $K_{d}$ to be determined, and its discrete form can be expressed as:

(1) $$u(k)=K_{p}err(k)+K_{i}\sum err(k)+K_{d}(err(k)-err(k-1))$$

where err(k) is the output error at time k of the system S in Figure 3. Let $\theta ={\left[K_{p},K_{i},K_{d}\right]}^{T}$ denote the vector of control parameters to be designed.

### 3.2. Outer Controller Design

Generally, when designing a control system, model identification and designing a controller are separated [25,26], but the theory of designing a control system considering the uncertainty of the system, revisiting the identification process and using it as a design process for the final control application is known as identification control (I4C). According to I4C theory, the best model in a control system may not be the one that provides the smallest output prediction error but the one that provides the best control performance for the closed-loop system. Therefore, this paper takes the prediction model as the design object and implements iterative optimization synchronously with the inner controller to find the best MPC prediction model. The inner PID and the controlled object constitute the system M. Its input is and its output is w, which can be expressed as:

(2) $$\left\{\begin{array}{c} \xi_{m}(t+1)=A\xi_{m}(t)+Bw(t)\\ y(t)=C\xi_{m}(t)+Dw(t) \end{array}\right.$$

where $\xi_{m}(t+1)$ represents the state of the system in the future. $\xi_{m}(t)$ represents the current state of the system. w(t) represents the system input. A,B,C and D represent the time-varying coefficients of the system M when the control result is optimal. This paper regards the predictive model of system M as a design parameter and finds the model that can provide the best closed-loop performance by solving the minimization problem shown below.

(3) $$\underset{w}{\min}\;J=Q_{y}\sum_{k=1}^{N_{p}}{\left(y(t+k|t)-y_{ref}(t+k)\right)}^{2}+Q_{w}\sum_{k=1}^{N_{p}}{\left(w(t+k|t)-w_{ref}(t+k)\right)}^{2}+Q_{u}\sum_{k=1}^{N_{p}}{\left(u(t+k|t)-u(t+k-1|t)\right)}^{2}$$

(4) $$\begin{array}{l} s.t.\;w(t+N_{u}+j|t)=w(t+N_{u}|t),j=1,\ldots ,N_{p}-N_{u}\\ \;y(t+k|t)=M(\mu ,w(t+k|t)),k=1,\ldots ,N_{p}\\ \;y_{\min}\leq y(t+k|t)\leq y_{\max}\\ \;w_{\min}\leq w(t+k|t)\leq w_{\max} \end{array}$$

where Δu(t+k|t)=u(t+k|t)−u(t+k−1|t). $N_{p}$ and $N_{u}$ represent the prediction time domain and the control time domain, respectively. $Q_{y},Q_{w}$ and $Q_{u}$ are positive definite matrices. $y_{ref}$ and $w_{ref}$ represent the reference values of the output and input of the system M, respectively. The model parameters μ include A,B,C and D.

### 3.3. V/G Monitoring Based on SAE-RF

The task of V/G monitoring based on SAE-RF is to estimate the V/G value online, according to the real-time input and output data of the controlled object system S and then compare it with the expected ${\left(V/G\right)}_{ref}$ value to design the controller. This design process requires compliance with the optimized performance function shown in (5) to obtain the optimal control input $w_{ref}$, which is used as the setting value for the outer MPC control output w.

(5) $$\underset{\Delta u}{\min}\;J^{*}=Q_{vg}\sum_{k=1}^{N}{\left(V/G(t+k)-{\left(V/G\right)}_{ref}(t+k)\right)}^{2}+Q_{\Delta w_{ref}}\sum_{k=1}^{N}{\left(w_{ref}(t+k)-w_{ref}(t+k-1)\right)}^{2}$$

where $Q_{vg},Q_{\Delta w_{ref}}$ are positive definite matrices.

In the actual CZ-SSCGP control process, if the quality of SSCs is to be guaranteed, V/G related to the crystal quality must always be in the expected control range. However, the complexity of the SSC growth environment and the confined nature of the CZ single crystal furnace make it difficult to obtain the axial temperature gradient at the solid–liquid interface directly by hard sensors, which in turn also makes it difficult to obtain V/G online [27]. Soft sensor modeling is an online estimation technique for dealing with unmeasurable variables. At present, the soft sensor model has been widely used in the field of industrial process control. Fortunately, a large amount of directly measurable data can be obtained from the actual CZ-SSCGP. Therefore, how to extract valuable information from these data and build an accurate V/G prediction model is a key problem to be studied.

**Figure 4:**
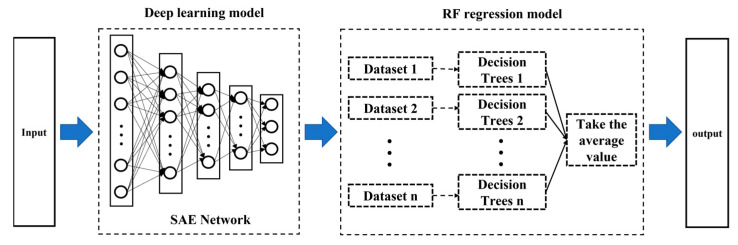
SAE-RF-based soft sensor model.

The actual CZ-SSCGP is a strongly nonlinear process. The intrinsic changes in the process in the actual process industry affect many process variables, and the dimensionality of the collected data is much larger than its actual dimensionality. At this point, some traditional linear soft sensor modeling methods can handle high-dimensional data problems, but such modeling methods perform poorly when solving such a strongly nonlinear problem for the CZ-SSCGP. As shown in Figure 4, in response to this problem, the SAE-RF soft sensor model is developed in this paper for real-time monitoring of V/G values, and its online estimation results are used in the V/G monitoring optimization link in (5). A stacked autoencoder (SAE) is a common deep learning model. This model is stacked by multiple autoencoders, and its purpose is to extract the high-level features of input data layer by layer. This process also reduces the input data dimension and converts complex input data into simple, high-level feature information. The SAE-RF-based soft sensor model extracts the deep features of the CZ-SSCGP through SAE’s deep learning model, and the obtained deep features are used as the input of RF (random forest), which in turn leads to the V/G prediction values.

The steps for the implementation of a soft sensor model based on predictive hierarchical control of SSC quality are as follows:

Step 1: Training the SAE-RF model.

Step 2: Give reference values ${\left(V/G\right)}_{ref}$, $y_{ref}$ and suitable closed-loop system parameters $N_{u},Q_{w}.Q_{y},Q_{u}$.

Step 3: Estimate the V/G value based on the input and output data of the system S at the previous moment.

Step 4: Solve the V/G deviation and obtain the best control input $w_{ref}$ of the system M through the V/G monitor.

Step 5: Optimally solve the best model parameters μ, θ and input w for the current system M.

Step 6: Determine the system M and solve the current output y of the system S.

Step 7: Return to Step 3.

## 4. Experiments and Results Analysis

**Figure 5:**
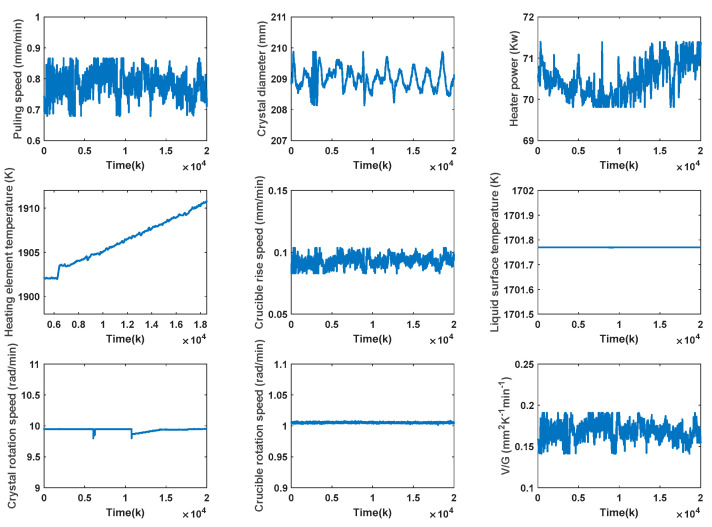
Historical data of each auxiliary variable and V/G values in the actual SSC production process.

**Figure 6:**
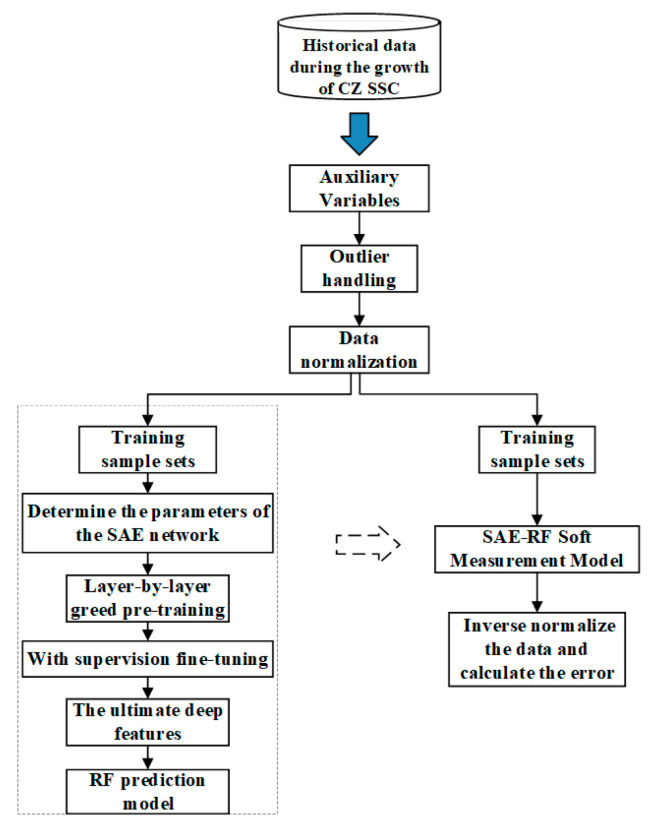
SAE-RF training process.

In this section, the training process of the proposed SAE-RF model selects measurable variables with a strong correlation with V/G, as auxiliary variables, as shown in Table 1. Figure 5 shows the historical data of each auxiliary variable and V/G values in the actual SSC production process. The offline training of the SAE-RF model is then performed according to the algorithmic flow shown in Figure 6.

**Figure 7:**
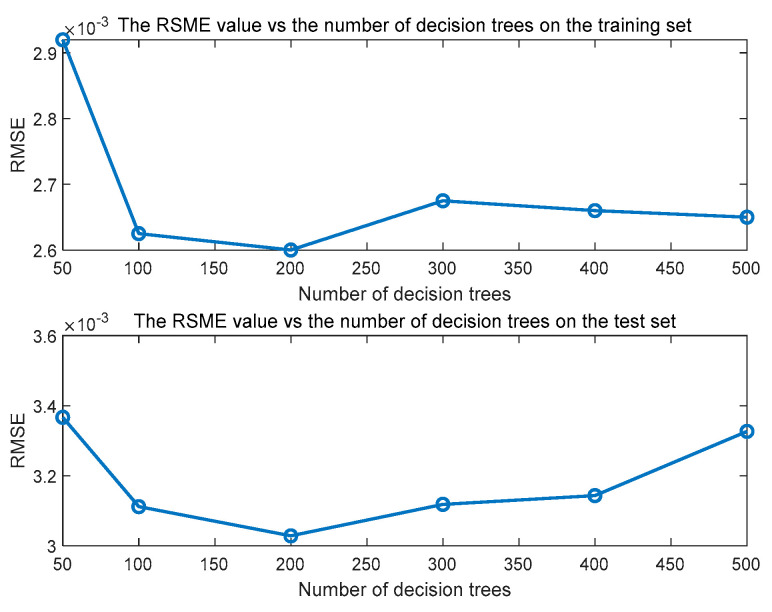
The relationship between the RSME of prediction error and the number of decision trees.

Next, the SAE network is trained layer by layer to determine the SAE network structure (8-5-3), weights and bias. During the RF training process, the features extracted from SAE are used as input to determine the RF network parameters. Considering the impact of the number of decision trees in RF, this paper uses the minimum prediction error between the predicted output of the SAE-RF and the actual value as the selection criterion. Figure 7 shows the prediction error evaluation index RMSE (root mean square error) of SAE-RF under different decision tree numbers. When the number of decision trees is 200, the RMSE index value of the prediction error is the smallest; thus, the number of decision trees is finally determined to be 200.

**Figure 8:**
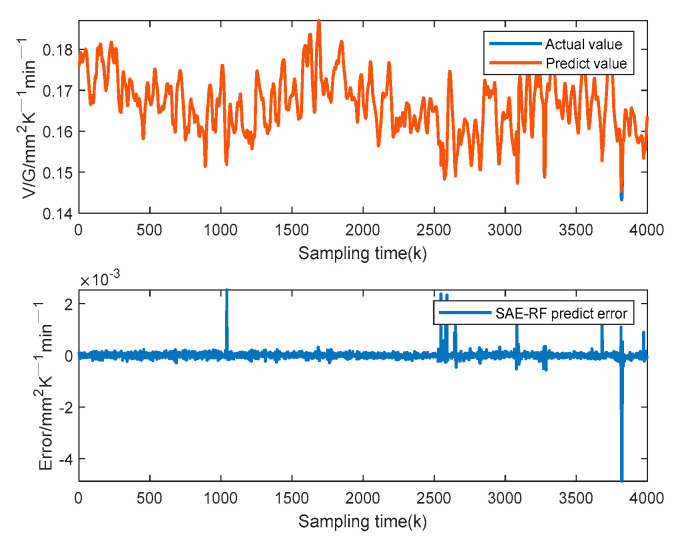
Prediction results of the proposed SAE-RF on the test sets.

It is worth noting that this paper uses a 3-fold cross-validation approach for model prediction performance evaluation in the offline modeling process to assist in selecting appropriate model parameters. This 3-fold cross-validation method is used by randomly dividing the training dataset into 3 parts, using 2 of them for training and the other one for validation. The process can be repeated 3 times, each time using different validation data. The prediction performance of the model is evaluated based on the average prediction error results after 3 modeling runs. Figure 8 shows the prediction result of the proposed SAE-RF model on the test set. The predicted values of V/G can track the trend of the actual values well. Therefore, it is known from Figure 7 and Figure 8 that the proposed prediction model SAE-RF has no overfitting or underfitting situations.

To test the control performance of the proposed SSC quality hierarchical predictive control method based on the soft sensor model (for convenience, the subsequent abbreviation is MPC-PID), the crystal diameter setting value is 209 mm, and the V/G setting value is 0.18 mm^2^ K^−1^ min^−1^. The outer MPC control parameter settings include $Q_{y}=0.3$, $Q_{w}=0.5$, $Q_{u}=0.6$. And $N_{p}=N_{u}=12$. The inner PID control parameter settings include $K_{p}=0.6865,K_{i}=-0.2672,\;K_{d}=-0.7752$. In addition, the gray wolf optimization algorithm (GWO) is used to solve the objective functions of (3) and (4). Furthermore, we compare the proposed method MPC-PID with conventional MPC and PID to analyze the crystal diameter tracking control performance and the V/G control output related to crystal quality.

**Figure 9:**
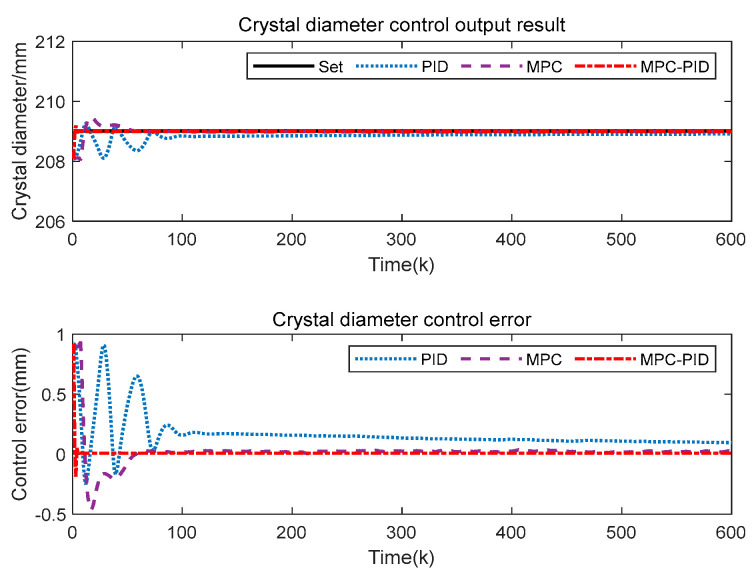
Crystal diameter control output results.

In Figure 9, the proposed MPC-PID hierarchical predictive control results are significantly better than those of MPC and PID. Specifically, the proposed MPC-PID hierarchical predictive control has a fast response speed, a small overshoot and a small steady-state tracking error. This is mainly because the inner layer PID in the proposed hierarchical predictive control strategy can quickly stabilize the control system, and the outer layer MPC can handle the uncertainty and physical constraints of the system well, thus enhancing the tracking control ability of the inner layer PID. This also means that the proposed MPC-PID hierarchical predictive control can ensure that the crystal diameter in the CZ-SSCGP can be controlled more accurately.

**Figure 10:**
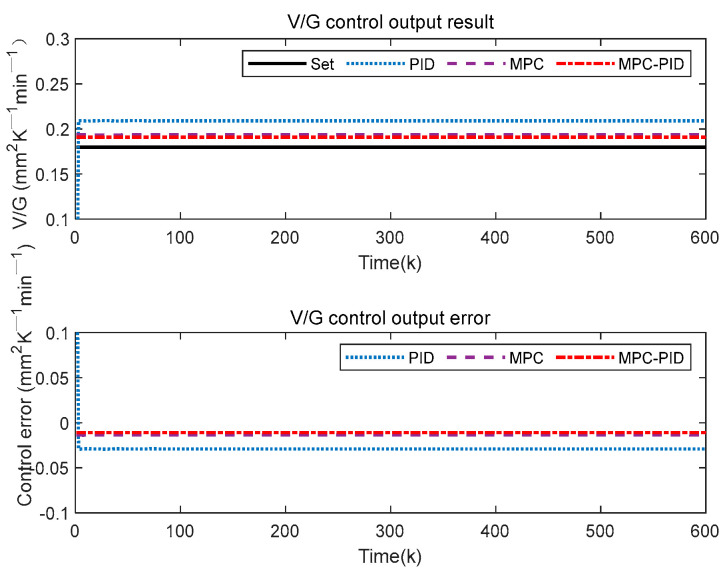
V/G value control output result.

Figure 10 shows the V/G value control output results of the proposed control method. Here, V/G is obtained by a soft sensor model based on SAE-RF. Compared with MPC and PID, the proposed MPC-PID hierarchical predictive control method can obtain the desired V/G monitoring results, which are closer to the set value. This result also shows that the proposed MPC-PID hierarchical predictive control method can ensure that crystal quality can be effectively controlled. However, the V/G output result of PID control is beyond the expected control range, which will lead to the production of SSC quality that cannot meet the actual industrial requirements. In addition, although the V/G output results of the MPC also meet the requirements, its crystal diameter control effect is worse than the proposed method. Therefore, from a comprehensive comparison, the proposed MPC-PID hierarchical predictive control method is more suitable for the SSC quality control of the CZ-SSCGP.

**Figure 11:**
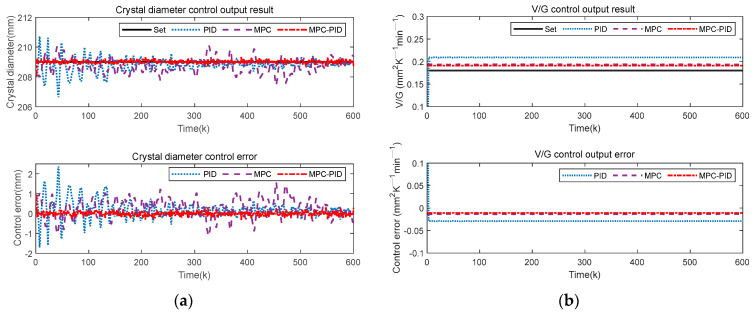
Control results of different control methods under Gaussian noise disturbance: (**a**) crystal diameter control output results; (**b**) V/G control output results.

**Figure 12:**
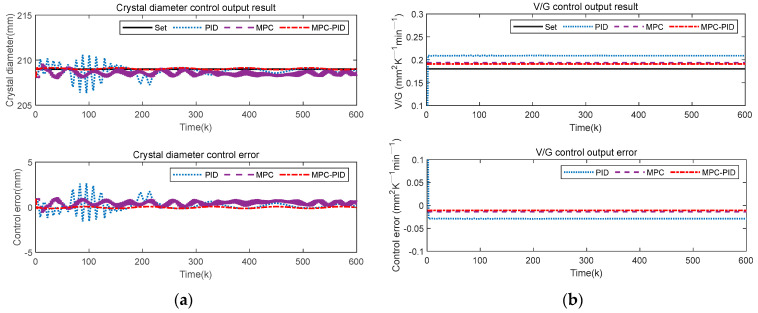
Control results of different control methods under sinusoidal noise disturbance: (**a**) crystal diameter control output results; (**b**) V/G control output results.

**Figure 13:**
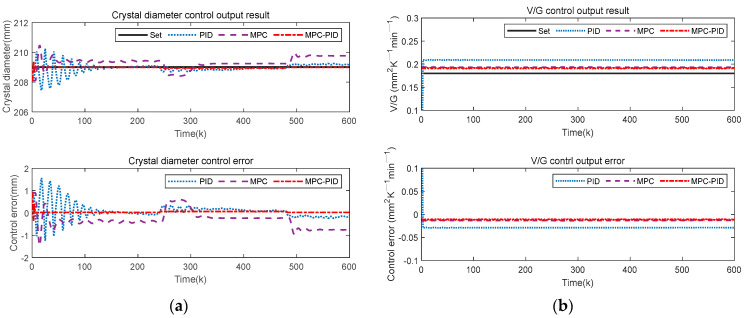
Control results of different control methods under square wave noise disturbance: (**a**) crystal diameter control output results; (**b**) V/G control output results.

Since there are many uncertainty disturbances in the actual CZ-SSCGP, the detection system generates random errors during the measurement process. Therefore, this paper added Gaussian random noise, sine wave noise and square wave noise to the output of the crystal growth system, respectively, to simulate the uncertainty noise mixed with sensor data acquisition. For this reason, Gaussian random noise, sinusoidal noise and square wave noise are added to the system output to test the robustness and stability of the proposed control method, respectively. Figure 11, Figure 12 and Figure 13 show the crystal diameter tracking results and V/G control output results under different disturbances, respectively.

Figure 11, Figure 12 and Figure 13 show that the PID control method is most obviously affected by different disturbances; that is, the crystal diameter output fluctuates greatly, and the V/G control output error is also large. In addition, although the MPC method is also affected by different disturbances, the crystal diameter tracking performance and V/G control output results are better than that of the PID. This means that MPC has good inherent robustness compared to PID. In short, compared with MPC and PID, the proposed MPC-PID hierarchical predictive control method has the best crystal diameter tracking results and the smallest V/G control output error. This further shows that the proposed hierarchical predictive control strategy based on the MPC-PID has strong robustness and can overcome the influence of different disturbances. In other words, the proposed control method can be well applied to the complex CZ-SSCGP and can ensure satisfactory control performance of crystal diameter and crystal quality.

To intuitively show the crystal diameter control performance and V/G control output performance of various control methods under different disturbances, Table 2, Table 3 and Table 4 give the maximum tracking error (MTE) of crystal diameter and V/G, and the corresponding RMSE value of the control error. Compared with MPC and PID, the proposed MPC-PID hierarchical predictive control method has the smallest MTE and RMSE. Specifically, taking Table 2 as an example, it can be intuitively seen that the tracking control performance of the proposed MPC-PID method is better than that of the PID and MPC methods, i.e., the proposed MPC-PID method has the smallest MTE and RMSE indexes. Specifically, compared to the PID and MPC methods, the proposed MPC-PID method reduces the crystal diameter control performance index (MTE) by 86% and 79%, respectively, while the RMSE index is also reduced by 80% and 76%, respectively. In addition, compared with the PID and MPC methods, the proposed MPC-PID method reduces the V/G control performance index MTE by 54% and 20%, respectively, and the RMSE index by 63% and 19%, respectively. In short, the comparison results of these control performance indexes further demonstrate that the proposed MPC-PID method has the best anti-disturbance control performance when the system is subject to uncertain external disturbances, while the PID method has a poor control effect.

In summary, according to the above various simulation experiments, the effectiveness of the SSC quality hierarchical prediction control method based on the soft sensor model is verified; that is, the crystal diameter and the V/G value related to the crystal quality can obtain a satisfactory control effect. In other words, the main reason for obtaining this control effect is that the inner PID control loop has good fast response performance and disturbance suppression, and the outer MPC control loop can handle the physical constraints of the system and optimize the control objectives in real time. In addition, the V/G monitoring module provides an accurate online estimation of V/G values related to crystal quality, which is used for optimal control of the outer MPC. It is worth mentioning that the MPC-PID hierarchical control strategy proposed in this paper can not only accurately track the target value of crystal diameter under the premise of ensuring crystal quality but also effectively suppressing the influence of external disturbances, thus obtaining good control performance and robustness.

## 5. Conclusions

In this paper, aiming at the problem of crystal quality control and uncertainty disturbance in the CZ-SSCGP, a soft sensor model-based crystal quality hierarchical prediction control method is proposed and used for the online monitoring of crystal diameter and crystal quality related V/G in CZ-SSCGP. The method includes an outer MPC controller, an inner PID controller and a V/G soft sensor model based on SAE-RF. The purpose of this control method is to achieve crystal diameter control while ensuring that crystal quality meets the desired target. According to various crystal quality control results, the proposed method has a more accurate crystal diameter control performance and satisfactory crystal quality control results than conventional MPC and PID control methods. In addition, the control test results under various disturbance conditions also show that the proposed method has good robust control performance, which can meet the goal of practical industrial production of high-quality semiconductor SSC. Although the proposed control method has many advantages, it also has some limitations, such as more controller parameters and time-consuming tuning. For this reason, our future work will be devoted to multivariate control of the CZ-SSCGP and optimization of the controller parameters.

## Figures and Tables

**Table 1:** Auxiliary variable.

Serial Number	Variable Name	Unit
1	crystal diameter	mm
2	main heater power	kW
3	crystal rise speed	mm/min
4	crucible rise speed	mm/min
5	heating element temperature	K
6	liquid surface temperature	K
7	crystal rotation speed	rad/min
8	crucible rotation speed	rad/min

**Table 2:** MTE and RMSE of control output error under Gaussian noise.

Method	Crystal Diameter (mm)		V/G (mm^2^ K^−1^ min^−1^)	
	MTE	RMSE	MTE	RMSE
PID	2.3627	0.5595	0.0247	0.0297
MPC	1.5723	0.4613	0.0141	0.0136
MPC-PID	0.3332	0.1092	0.0113	0.0110

**Table 3:** MTE and RMSE of control output error under sinusoidal noise.

Method	Crystal Diameter (mm)		V/G (mm^2^ K^−1^ min^−1^)	
	MTE	RMSE	MTE	RMSE
PID	2.6453	0.5655	0.0298	0.0267
MPC	1.1055	0.5334	0.0139	0.0136
MPC-PID	0.1985	0.0891	0.0113	0.0109

**Table 4:** MTE and RMSE of control output error under square wave noise.

Method	Crystal Diameter (mm)		V/G (mm^2^ K^−1^ min^−1^)	
	MTE	RMSE	MTE	RMSE
PID	1.5552	0.4840	0.0295	0.0297
MPC	1.4537	0.3193	0.0138	0.0134
MPC-PID	0.3584	0.0623	0.0122	0.0112

## Data Availability

The research data is confidential in the industry. Due to the principle of confidentiality, the data cannot be shared. Please understand.
